# Integrin α6β4 Upregulates PTPRZ1 Through UCHL1-Mediated Hif-1α Nuclear Accumulation to Promote Triple-Negative Breast Cancer Cell Invasive Properties

**DOI:** 10.3390/cancers16213683

**Published:** 2024-10-31

**Authors:** Min Chen, Parvanee A. Karimpour, Andrew Elliott, Daheng He, Teresa Knifley, Jinpeng Liu, Chi Wang, Kathleen L. O’Connor

**Affiliations:** 1 Markey Cancer Center, University of Kentucky, Lexington, KY 40536, USA; parvanee.karimpour@uky.edu (P.A.K.); andrews.elliott@uky.edu (A.E.); dhe2@g.uky.edu (D.H.); teresa.knifley@uky.edu (T.K.); jinpeng.liu@uky.edu (J.L.); chi.wang@uky.edu (C.W.); 2 Department of Toxicology and Cancer Biology, University of Kentucky, Lexington, KY 40536, USA; 3 Department of Molecular and Cellular Biochemistry, University of Kentucky, Lexington, KY 40536, USA; 4 Division of Cancer Biostatistics, Department of Internal Medicine, University of Kentucky, Lexington, KY 40536, USA

**Keywords:** integrin alpha6beta4, basal breast cancer, triple negative breast cancer, hypoxia response, protein tyrosine phosphatase

## Abstract

Integrin α6β4 drives triple-negative breast cancer (TNBC) aggressiveness through the transcriptional regulation of key genes. Here, we investigated how integrin α6β4 regulates protein tyrosine phosphatase receptor type Z1 (PTPRZ1). Using stable re-expression of integrin β4 (ITGB4) in cells naturally devoid of integrin α6β4 or knockdown or knockout (KO) of ITGB4, we found that integrin α6β4 regulates PTPRZ1 expression. To gain mechanistic insight, we focused on Hif-1α due to the impact of integrin α6β4 on a hypoxia-associated signature. We found that nuclear localization of Hif-1α, but not Hif-2α, was substantially enhanced with integrin α6β4 signaling. Hif-1α knockdown by shRNA or chemical inhibition decreased PTPRZ1 expression, while chemical activation of Hif-1α increased it. Upstream of Hif-1α, integrin α6β4 upregulates UCHL1 to stabilize Hif-1α and ultimately regulate PTPRZ1. Inhibition of UCHL1 and PTPRZ1 dramatically decreases integrin α6β4-mediated cell migration and three-dimensional invasive growth. Finally, public breast cancer database analyses demonstrated that ITGB4 correlates with PTPRZ1 and that high expression of ITGB4, UCHL1, HIF1A, and PTPRZ1 associated with decreased overall survival, distant metastasis free survival, post progression survival, and relapse-free survival. In summary, these findings provide a novel function of integrin α6β4 in promoting tumor invasive phenotypes through UCHL1-Hif-1α-mediated regulation of PTPRZ1.

## Simple Summary

Integrin α6β4 makes triple-negative breast cancer (TNBC) more aggressive by controlling genes that drive tumor invasion and metastasis. PTPRZ1 is linked to cancer relapse, but its role in TNBC is not well understood. We discovered that PTPRZ1 expression is significantly increased by integrin α6β4 in TNBC. We also found that integrin β4 gene expression correlates with PTPRZ1 in breast cancer patient samples. Importantly, we found that integrin α6β4 controls PTPRZ1 through UCHL1, which in turn stabilized Hif-1α. When UCHL1 or PTPRZ1 are blocked, the aggressiveness driven by integrin β4 signaling decreased substantially. Data analysis showed that high levels of these genes (ITGB4, UCHL1, Hif1α, PTPRZ1) are linked to worse survival rates, especially in patients who received chemotherapy treatment. Our findings suggest that integrin α6β4 helps TNBC become invasive through UCHL1-Hif-1α regulation of PTPRZ1, pointing to a new approach to targeting integrin α6β4 signaling using PTPRZ1 and UCHL1 inhibitors.

## 1. Introduction

Triple negative breast cancer (TNBC) accounts for 15–20% of breast cancer cases and 30% of breast cancer deaths due to its aggressive behavior. Notably, about 50% of patients with early-stage TNBC will develop recurrence, and 37% of patients will succumb to their disease within the first five years after surgery [1]. Investigating and understanding the molecular mechanisms that drive these aggressive properties are necessary to inform new treatment strategies for TNBC patients.

Integrin α6β4 expression is elevated in over 80% of TNBC cases [2]. As a laminin receptor, integrin α6β4 binds the basement membrane and mediates hemidesmosome formation to maintain epithelium integrity in normal physiology. During tumor progression, integrin α6β4 is liberated from these large hemi-desmosomal plaques, where it coordinates and amplifies signals from the microenvironment to promote an invasive and metastatic phenotype in several types of cancer, including breast cancer [3,4]. Integrin α6β4 activates tumor progression-associated signaling pathways by cooperating with growth factor receptors, such as EGFR and c-Met, as well as altering the transcriptome through transcription factors, such as AP1, NF- κB, and NFATs, in conjunction with epigenetic changes [1]. Bierie et al. [5] reported that integrin β4 can be used to enable the stratification of mesenchymal-like TNBC that could serve as a source of relapse. Furthermore, they found that, among patients being treated with chemotherapy, elevated levels of integrin β4 expression was associated with a worse five-year probability of relapse-free survival [5]. While the role of integrin α6β4 in promoting tumor cell survival, proliferation, and migration is well-established, recent studies on the impact of its signaling on TNBC therapeutic response are conflicting [6,7]. This suggests that further exploration of how integrin α6β4 signaling can be targeted will enable viable strategies for personized medicine in TNBC treatment and improve patient outcomes.

Protein tyrosine phosphatase receptor type Z1 (PTPRZ1, also known as PTPζ or PTPβ), belongs to the R5 sub-family of receptor-type protein tyrosine phosphatases and is a single-pass type I membrane protein. PTPRZ1 is preferentially expressed in the central nervous system and has been suggested to have a close association with cancer stemness in human glioblastoma, as demonstrated by altering the expression of stem cell transcription factors following knockdown of PTPRZ1 [8]. Pleiotrophin (PTN) is a well-accepted ligand for PTPRZ1. In glioma, the PTN-PTPRZ1 axis promotes malignant growth through paracrine signaling [8], thus suggesting PTN-PTPRZ1 is a viable target in glioma [9,10]. The biological function of PTPRZ1 has been demonstrated in various cancers, such as small cell lung cancer, cervical carcinoma, oral squamous cell carcinoma, and gastric cancer [11]. Notably, the progression of these cancers is driven by integrin α6β4 [1]. Several agents that target PTPRZ1 have been developed, including anti-PTPRZ1 antibodies, inhibitors such as MY10, MY33-3NAZ2329, SCB4380, and protamine [8,11]. However, select studies have implicated PTPRZ1 as a tumor suppressor [11,12] and low PTPRZ1 expression was found to be linked to a worse response to c-Met tyrosine kinase inhibitor in lung adenocarcinoma [13]. PTPRZ1 activators, such as rebamipide, have been used to activate PTPRZ1 in PTPRZ1–downregulated cancer cells. In breast cancer, PTPRZ1 and PTN are highly expressed in relapsed as well as chemo-resistant TNBC [14], and PTPRZ1 is considered as an independent risk indicator for TNBC recurrence and metastasis [15]. However, the biological functions of PTPRZ1 and how it is regulated in TNBC are not clear.

Ubiquitin C-terminal hydrolase 1 (UCHL1) is an enzyme unique for its ligase and hydrolase activities. Although UCHL1 was first detected as a “brain-specific protein” [16], its expression is not limited to the neuronal compartment. UCHL1 is also highly expressed in carcinomas of various tissue origins, including those from brain, lung, breast, kidney, colon, prostate, pancreas, and mesenchymal tissues. Loss-of-function studies and an inhibitor for UCHL1 confirmed the importance of UCHL1 for cancer therapy [17]. UCHL1 plays critical roles in various biological processes, including spermatogenesis, oncogenesis, angiogenesis, cell proliferation and differentiation in skeletal muscle, inflammation, tissue injury, neuronal injury, and neurodegeneration [18]. UCHL1 has been shown to regulate protein stabilities, including EGFR and Hif-1α [17,19]. In this study, we implicate UCHL1-mediated Hif-1α nuclear accumulation in integrin α6β4-regulated PTPRZ1 expression that drives the invasive phenotype of TNBC cells.

## 2. Materials and Methods

### 2.1. Cell Lines and Drug Treatments

All cells were obtained from American Type Culture Collection (Manassas, VA, USA). BT549 cells were cultured in RPMI1640 (ThermoFisher Scientific, Waltham, ME, USA) containing 10 µg/mL insulin (MilliporeSigma, St. Louis, MO, USA). MDA-MB-231 cells were maintained in DMEM-F12 (ThermoFisher Scientific). BT549 cells that stably expressed integrin β4 (BT549-β4) or empty vector (BT549-EV) were generated as described previously [20]. SUM149 cells were cultured in high-glucose DMEM (ThermoFisher Scientific) with 5% FBS, 5 µg/mL insulin and 1 µg/mL hydrocortisone. HMLE cells were cultured in MEBM™ Mammary Epithelial Cell Growth Basal Medium (Lonza, Morristown, NJ, USA) supplemented with 5% FBS, 30 µg/mL bovine pituitary extract, and 20 ng/mL hEGF, 0.5 µg/mL Hydrocortisone, GA-1000 (1:1000) and 10 µg/mL insulin. All supplements were from Lonza. All media were supplemented with 10% FBS (MilliporeSigma) unless otherwise specified, 1% L-glutamine, 1% penicillin and 1% streptomycin (ThermoFisher Scientific). All cell lines, including all derivative cells (ITGB4 CRISPR/Cas9 gene editing and retroviral expression), were authenticated by Laboratory Corporation of America Holdings (LabCorp, Burlington, NC, USA) using short tandem repeat (STR) profiling and were confirmed as mycoplasma free. LDN-57444 and NAZ2329 were purchased from MedChemExpress (Monmouth Junction, NJ, USA), and PX-478 was purchased from Selleckchem (Houston, TX, USA). All inhibitors at the indicated concentrations were added to cells under normal culture conditions.

### 2.2. shRNA Stable Cell Line Generation

Lentiviral-based shRNA (Sigma Aldrich, St. Louis, MO, USA) was used to generate stable knockdown of target genes in select cell lines, as described previously [21]. Lentivirus was produced by combining MISSION constructs for packaging (psPAX2), envelope (pDM2G) vectors, and targeting shRNA or a non-targeting vector (pLKO.1) at a 4:2:1 ratio. Poly-ethylenimine (PEI; Polysciences, Warrington, PA, USA) was combined with the DNA mixture at a DNA to PEI ratio of 3:1 and added to 70% confluent HEK293LTV cells that had been passaged 24 h prior. Conditioned media was collected 36 h post transfection, centrifuged at 2000× *g* for 10 min and viral supernatant was filtered with 0.45 µm filters. Cells were passaged to 70% confluence in 10 cm dishes and 4 mL of viral supernatant added in combination with 8 μg/mL hexadimethrine bromide (polybrene, Sigma Aldrich). Cells were placed under puromycin selection (2 μg/mL) for a minimum of 7 days, or until cell death stopped due to selection. Gene expression was measured by qPCR or immunoblotting 24 h following removal of puromycin. Sequences for shRNA for UCHL1 shRNA are: #1 GTGTGAGCTTCAGATGGTGAA, #2 CGGGTAGATGACAAGGTGAAT, #3CTGTGGCACAATCGGACTTAT, #4 CCAGCATGAGAACTTCAGGAA, and #5 CAGTTCTGA-AACAGTTTCTTT. Sequences for shRNAs for HIF1A are: #1 CCGCTGGAGACACAAT-CATAT, #2 CCAGTTATGATTGTGAAGTTA, and #3 GTGATGAAAGAATTACCGAAT.

### 2.3. Three-Dimensional (3D) Culture

As described previously [21], 3D culture for BT549 cells was performed by seeding cells (5 × 10^3^) in 200 μL RPMI plus 2% FBS onto solidified growth factor reduced Matrigel (BD Biosciences, Franklin Lakes, NJ, USA; 100 μL per well of 8-well chamber slide) and then covered with 10% Matrigel containing medium. The next day, LDN57444 and NAZ2329 at indicated concentrations were added to the cultures. When the control cells developed an invasive growth phenotype (6–8 days), phase contrast images of randomly chosen fields were taken with a Nikon Ti-E inverted microscope (Konan, Minato-ku, Tokyo, Japan) using Nikon Elements software (NIS-Elements AR 5.00.00). The diameter of each colony within a field was measured and the volume of the colonies and the percentage of colonies with invasive growth were calculated, as described previously [22].

### 2.4. Quantitative Real-Time Polymerase Chain Reaction (qPCR)

Total RNA (1 μg, extracted from cells using Trizol reagent) was used to prepare cDNA using the high-capacity cDNA Reverse Transcription Kit (Applied Biosystems, Waltham, MA, USA). Target gene expression was then assessed by comparative Ct (∆∆Ct) using commercially available probes and master mix reagent and performed on a StepOnePlusTM 96-well instrument as described by the manufacturer (Applied Biosystems). The expression level of each gene was normalized by 18S RNA and reported as a relative level to a specified control.

### 2.5. Subcellular Protein Fractionation and Immunoblot Analysis

Cells, as noted, were lysed in buffer with phosphatase inhibitors (20 mM Tris-HCl, pH 7.5, 150 mM NaCl, 1 mM Na_2_EDTA, 1 mM EGTA, 1% Triton X-100, 2.5 mM sodium pyrophosphate, 1 mM β-glycerophosphate, 1mM sodium orthovanadate, 1 µg/mL leupeptin, 1 mM PMSF), sonicated, and processed for subcellular fractionation using the Subcellular Protein Fractionation Kit (ThermoFisher Scientific) according to the manufacturer’s instructions. For whole cell lysate assessment, cells were harvested with RIPA buffer (150 mM NaCl, 0.5 mM EGTA, 0.5% sodium deoxycholate, 0.1% SDS, 1% Triton X-100, 50 mM Tris-HCl pH 7.4, 15 µg/mL protease inhibitor cocktail (Millipore Sigma), 1 mM PMSF, 50 mM NaF and 10 mM sodium pyrophosphate). Fractionated or whole cell lysates (80 μg), as noted, were separated by 8% or 15% SDS–PAGE and then immunoblotted with various antibodies: integrin β4 (H101, cat# sc-9090), UCHL1 (C-4, cat# sc-271638, Santa Cruz, Biotechnology, Dallas, TX, USA), PTPRZ1 (cat# HPA015103, Millipore Sigma), Hif-1α (D2U3T, cat# 14179) and Hif-1β (D28F3, cat #5537) (Cell Signaling Technology, Danvers, MA, USA), and Hif-2α (cat# ab199, Abcam, Waltham, MA, USA). β-actin (MilliporeSigma) was used as a loading control for total cell lysates, tubulin (MilliporeSigma) for cytosolic fraction, and p84 (5E10, cat# GTX70220, GeneTex, Irvine, CA, USA) for nuclear extracts.

### 2.6. MTT Assay

Cells (2 × 10^3^) were seeded in each well of the 96-well plates the day before treatments as noted. MTT assays were performed in triplicate or greater, as reported previously [7]. Briefly, 20 µL MTT (5 mg/mL) was added to each well and incubated at 37 °C for 3 h. To dissolve the formazan precipitate, 100 µL of stop mix solution containing 90% isopropanol and 10% DMSO was added. OD 570 was read and recorded every day for a 6-day period.

### 2.7. Migration Assay

Transwell chemotactic migration assays were performed as described previously [22]. Briefly, cells were left untreated or treated with 5 μM LDN57444 or 10 µM NAZ2329 for 2 hrs, then cells (5 × 10^4^) were placed into the top of 15 μg/mL of laminin-1-coated Transwells (6.5 mm diameter, 8 µm pore size; Corning, Corning, NY, USA) and allowed to migrate for 4 hrs toward serum-free media containing 250 µg/mL BSA or 5 ng/mL EGF in the absence or presence of LDN57444 or NAZ2329, as noted. Non-migrated cells were then removed from the upper chamber using a cotton swab and migrated cells were fixed, stained with 1% crystal violet, and counted using an inverted microscope. Four fields per well were counted and averaged, and the data were presented as the mean number of cells invaded per mm^2^ ± standard deviation (SD) from triplicate determinations. Data reported are representative of at least three separate experiments.

### 2.8. Fluorescence-Activated Cell Sorting (FACS) of Live Cells

Cells were prepared for FACS analysis by trypsinization and quenching with media containing 10% FBS. Cells were collected in FACS media containing 1% FBS, 5 mM EDTA, and 1 g/mL glucose. The suspended cells were then incubated with or without efluor 660 conjugated anti-human CD104 (integrin β4 subunit) antibody (439-9B, cat# 50-1049-80, eBioscience, San Diego, CA, USA) at 1:500 dilution for 1 hr at 4° C and washed three times with PBS containing 0.25 mg/mL heat inactivated BSA (PBS/BSA). Cells were then analyzed and sorted on a BD FACSymphony S6 Cell Sorter in the Flow Cytometry and Immune Monitoring Shared Resource Facility at the University of Kentucky Markey Cancer Center.

### 2.9. Database Analysis

To correlate integrin α6β4 and PTPRZ1 expression in clinical breast cancer samples, we accessed published patient-level genomic and clinical data from the TCGA PanCancer Atlas Breast Invasive Carcinoma dataset via cBioPortal (www.cbioportal.org, accessed on 15 July 2024) database. Spearman and Pearson correlation coefficients and *p*-values for integrin α6β4 (ITGB4) and PTPRZ1 expression for breast cancer samples and basal-like samples were calculated in cBioPortal. Scatter plots of ITGB4 and PTPRZ1 expression were plotted in BioRender (mRNA expression, log2(value+1), RSEM, batch normalized from Illumina HiSeq_RNASeqV2). To assess PTPRZ1 expression across cancer types, we downloaded expression data from all available TCGA studies in cBioPortal using the TCGA PanCancer Atlas Studies Quick Select feature. mRNA expression data from representative cancers were plotted in BioRender (log2(value+1), RSEM, batch normalized from Illumina HiSeq_RNASeqV2). Likewise, the Spearman correlations between ITGB4 and PTPRZ1 expression across multiple cancer types and within breast cancer subtypes were calculated in cBioPortal using the aforementioned TCGA datasets, and select correlations were represented in table format. To determine the impact of treatment on pathway expression in breast cancer samples, we downloaded clinical and genomic data from The Metastatic Breast Cancer Project (Provisional, December 2021) using cBioPortal. Treatment status (PATH sample treatment naive) vs mRNA expression (mRNA Seq V2 RSEM, log2(value+1)) was plotted in BioRender. Using the Kaplan–Meier (KM) Plotter database breast gene chip dataset (www.kmplot.com, accessed on 1 September 2024), we assessed the prognostic value of the integrin α6β4-UCHL1-Hif-1α-PTPRZ1 pathway on survival outcomes in breast cancer. Based on the mean expression levels of ITGB4, UCHL1, HIF1A, and PTPRZ1, all patients were divided into high and low expression groups using selected optimal cutoff values. Survival analysis plots, hazard ratios (HR), 95% confidence intervals (CI), and log-rank *p*-values were calculated using KM Plotter. For survival analysis, we calculated overall survival (OS, n = 1880), distant metastasis-free survival (DMFS, n = 2767), relapse-free survival (RFS, n = 4934), and post-progression survival (PPS, n = 458) for breast cancer. We then further assessed the impact of systemic therapy on RFS in breast cancer between high and low expression of the integrin α6β4-UCHL1-Hif1α-PTPRZ1 axis (systemic therapy n = 2573, no systemic therapy n = 1025).

### 2.10. Statistical Analysis

Data were compared and analyzed using a two-tailed or one-tailed unpaired Student’s *t*-test. All in vitro experiments were performed at least three times and the representative data were shown and presented as mean ± SD, unless stated otherwise. *p* values < 0.05 between groups were considered significantly different.

## 3. Results

### 3.1. Integrin α6β4 Regulates the Expression of PTPRZ1 in Breast Cancer Cells and Their Expression Is Correlated in Breast Cancer Patients

**Figure 1:**
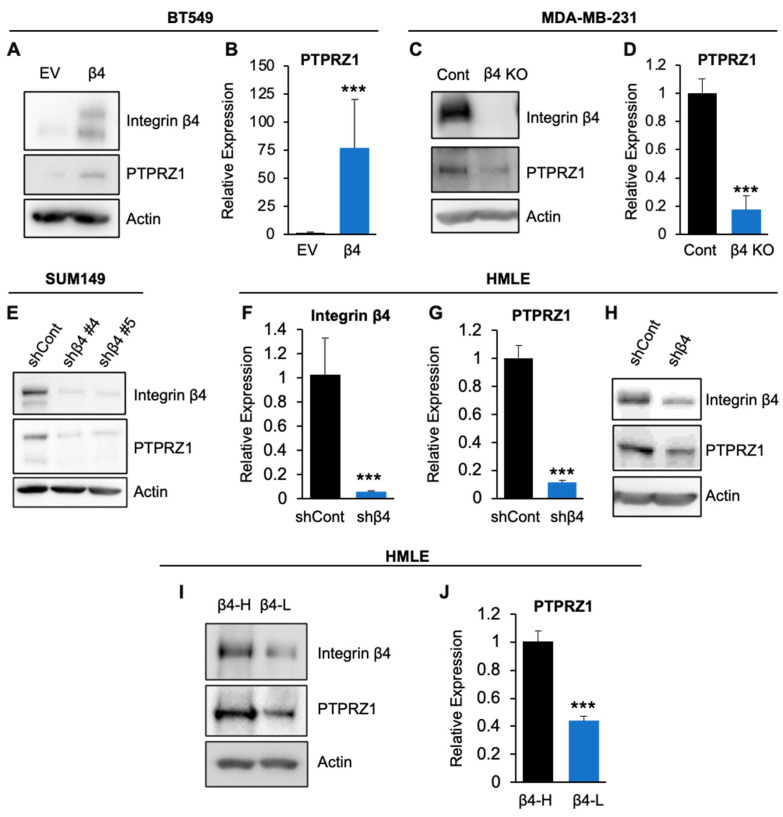
Integrin α6β4 regulates the expression of PTPRZ1 in breast cells. BT549 cells expressing empty vector (EV) or wildtype integrin β4 (**A**,**B**), MDA-MB-231 cells (parental, control) or integrin β4 knockout (KO) (**C**,**D**), SUM149 (**E**) and HMLE (**F**–**H**) cells with integrin β4 shRNA knockdown (shβ4) or control shRNA (shCont), and (**I**,**J**) HMLE cells sorted for integrin β4 high expression (β4-H) and low expression (β4-L) were assessed for the expression of indicated genes by immunoblotting (**A**,**C**,**E**,**H**,**I**) or qPCR (**B**,**D**,**F**,**G**,**J**). *** *p* < 0.0001.

We reported previously that PTPRZ1 is upregulated by integrin α6β4 in MDA-MB-435 cells [22]. However, the cell signaling that regulates PTPRZ1 expression downstream of integrin α6β4 and how this regulation contributes to breast cancer invasive phenotype was not explored. By RNA sequencing analysis on a more relevant TNBC cell model, we identified that exogenous integrin β4 expression elevated the transcript levels of PTPRZ1 by up to 84-fold in BT549 cells as determined by RNA-seq. This observation was confirmed using immunoblot and qPCR analyses (Figure 1A,B). Using CRISPR-Cas9 editing, we found that knockout of integrin β4 in MDA-MB-231 cells significantly downregulated PTPRZ1 expression (Figure 1C,D). Targeting integrin β4 by shRNA demonstrated that integrin α6β4 also regulated PTPRZ1 expression in SUM149 (Figure 1E) and HMLE cells (Figure 1F–H). We also sorted HMLE cells into high and low integrin β4 population by FACS analysis. Immunoblotting analysis revealed that HMLE β4 high (β4-H) cells expressed high levels of PTPRZ1 compared to β4 low (β4-L) cells (Figure 1I). qPCR results showed that β4 high cells express about 2-fold higher levels of PTPRZ1 compared to the low β4 cells population (Figure 1J), which is in agreement with published RNA-seq data [5]. These data collectively demonstrate that PTPRZ1 is regulated by integrin α6β4 in breast cells.

**Figure 2:**
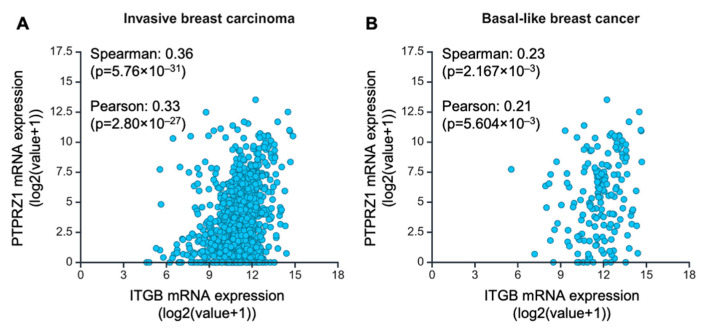
Integrin α6β4 expression correlates with PTPRZ1 expression in breast cancer. Patient-level genomic and clinical data from TCGA PanCancer Atlas Breast Invasive Carcinoma dataset demonstrated a correlation between integrin β4 (ITGB4) and PTPRZ1 expression in (**A**) invasive breast carcinoma and (**B**) basal-like breast cancer (mRNA expression, log2(value+1), RSEM, batch normalized from Illumina HiSeq_RNASeqV2). Spearman and Pearson correlation coefficients and *p* values for ITGB4 and PTPRZ1 expression were calculated in cBioPortal.

To examine the clinical association of PTPRZ1 with integrin β4 (ITGB4) in breast cancer, we assessed the correlation of ITGB4 and PTPRZ1 by analyzing the data from the TCGA PanCancer Atlas Breast Invasive Carcinoma Dataset. We found that ITGB4 expression correlated with PTPRZ1 in tumor samples from patients with both invasive breast carcinoma (Figure 2A) and basal-like breast cancer (Figure 2B). Interestingly, ITGB4 also significantly correlated with PTPRZ1 in breast cancer Luminal A subtype, but not Luminal B or Her2 breast cancer subtypes (Table 1). Further analyzing TCGA PanCancer Atlas data, we confirmed that, in addition to breast cancer, PTPRZ1 is highly expressed in various cancer types, such as glioma, prostate, lung, esophageal, and select neuroendocrine tumors (Figure S1). Notably, integrin β4 expression exhibits significant positive correlation with PTPRZ1 expression in most of these cancer types with the exception of glioma (Table S1), which uniformly displayed the highest level of PTPRZ1. These data support our findings on the association of integrin β4 and PTPRZ1 using cell lines, extend these findings to other cancer types, and suggest the importance of integrin β4-regulated PTPRZ1 in cancer progression.

### 3.2. Integrin β4 Promotes Hif-1α Nuclear Accumulation and Hif-1α Is Critical for Integrin β4-Mediated PTPRZ1 Upregulation

**Figure 3:**
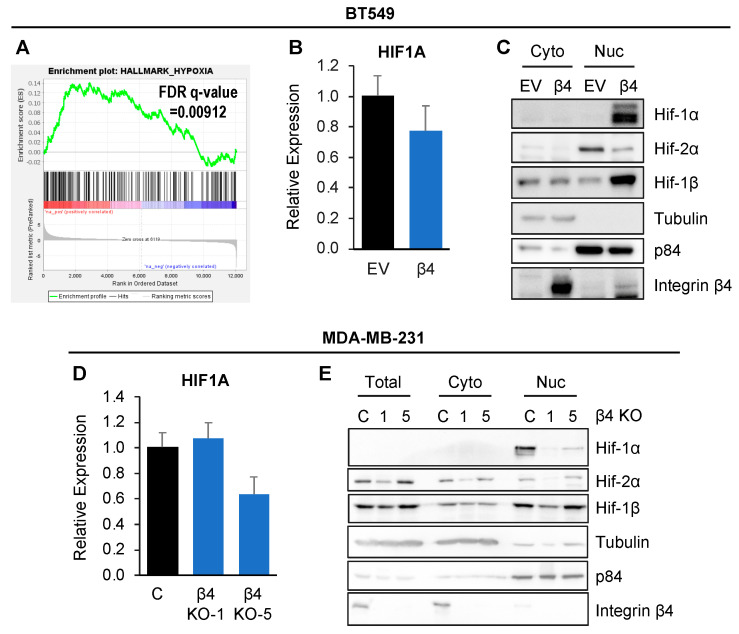
Integrin α6β4 promotes Hif-1α nuclear accumulation. (**A**) Hypoxia enrichment plot showing the impact of integrin α6β4 on BT549 cells. (**B**) qPCR for HIF1A in BT549-EV and BT549-β4 cells. (**C**) Immunoblot of cytosolic and nuclear BT549 extracts for indicated proteins. (**D**) qPCR for HIF1A in MDA-MB-231 cells and representative β4 KO clones using two distinct guide RNAs. (**E**) Immunoblot analysis of total, cytosol and nuclear extracts from cells in (**D**) for indicated proteins. Tubulin was used as the marker for total protein, and p84 was used to mark nuclear fractions.

Hypoxic stress can regulate PTPRZ1 expression [23]. Additionally, PTPRZ1 was identified as a responsive gene of Hif-2α and regulated by Hif-2α but not Hif-1α in HEK293T and Hep3B [23,24]. Gene ontology (GO) analysis of RNA-seq data in BT549 cells suggested that integrin β4 positively correlated with genes in the hypoxia pathway (Figure 3A). Accordingly, we tested if integrin α6β4 impacts HIF1A expression levels by qPCR and cell fractionation. We found that β4 integrin did not change HIF1A expression at the mRNA level (Figure 3B) in BT549 β4 cells. However, Hif-1α and Hif-1β accumulated in the nuclear compartment of BT549 β4 cells, while the level of Hif-2α slightly decreased (Figure 3C). Similar results were obtained in MDA-MB-231 cells with integrin β4 knockout (Figure 3D). EPAS1, the gene that encodes Hif-2α, was significantly decreased at the mRNA level in RNA sequencing data in BT549 β4 cells compared to BT549 EV cells. In contrast, knockout of integrin β4 in MDA-MB-231 cells decreased the nuclear levels of Hif-1α and Hif-1β while Hif-2α levels were inconsistent among KO clones (Figure 3E). Therefore, we focused on Hif-1α for further investigation.

**Figure 4:**
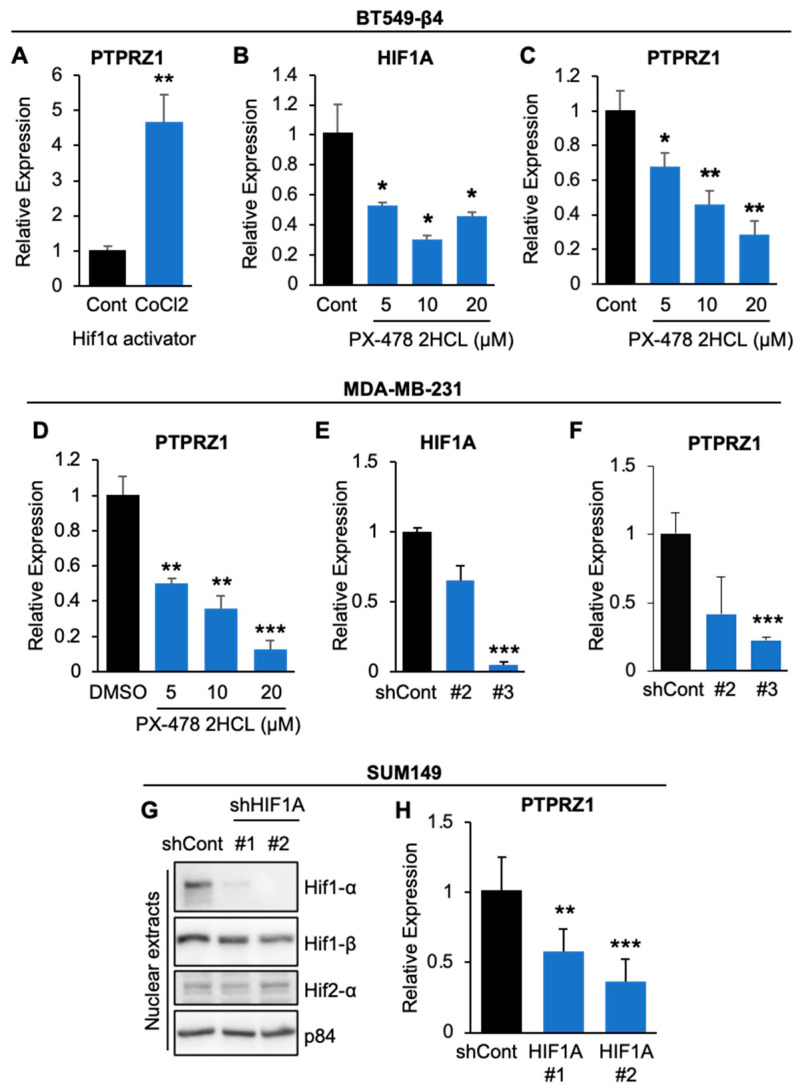
Hif-1α is required for PTPRZ1 expression downstream of integrin α6β4**.** BT549-β4 cells (**A**–**C**) were treated with 100 µM CoCl_2_, a Hif-1α activator (**A**), or indicated doses of Hif-1α inhibitor PX-478. After 48 hrs, qPCR was performed to assess the expression of PTPRZ1 (**A**,**C**) or HIF1A (**B**). (**D**–**F**) MDA-MB-231 cells were treated with indicated doses of PX-478 for 72 h (**D**) or HIF1A was stably knocked down using shRNA (**E**,**F**) and then assessed for PTPRZ1 (**D**,**F**) and HIF1A (**E**) by qPCR. (**G**,**H**) SUM149 cells with HIF1A knockdown by two different shRNAs were immunoblotted for the indicated proteins (**G**) or PTPRZ1 expression by qPCR (**H**). * *p* < 0.05, ** *p* < 0.005, *** *p* < 0.0001.

Next, we assessed whether integrin β4-mediated Hif-1α accumulation regulated PTPRZ1 expression using multiple cell lines. Since PTPRZ1 expression in BT549 EV cells is low and under the detection limit in most instances, we focused on BT549 β4 cells to further address whether active Hif-1α plays an important role in PTPRZ1 regulation. First, we treated BT549 β4 cells with 100 µM CoCl_2_, a Hif-1α activator, for 24 h, then performed qPCR for PTPRZ1. We found that CoCl_2_ treatment induced about a 4-fold increase in PTPRZ1 expression in BT549 β4 cells (Figure 4A). Next, we treated BT549 β4 (Figure 4B,C) and MDA-MB-231 cells (Figure 4D) for 72 hrs with Hif-1α inhibitor PX-478 at 5 µM, 10 µM and 20 µM prior to assessing expression levels of HIF1A (Figure 4B) and PTPRZ1 (Figure 4C,D) by qPCR and demonstrated that PTPRZ1 expression was reduced with inhibition of Hif-1α. To further define whether the effects of PX-478 were specific to Hif-1α, we knocked down HIF1A in MDA-MB-231 (Figure 4E,F) and SUM149 (Figure 4G,H). Effective knockdown of HIF1A in MDA-MB-231 (Figure 4E) and SUM149 (Figure 4G) cells decreased PTPRZ1 expression as determined by qPCR analysis (Figure 4F,H). Collectively, these data demonstrate that integrin β4 upregulates PTPRZ1 through Hif-1α.

### 3.3. Integrin β4-Mediated Upregulation of UCHL1 Contributes to Hif-1α Stability and Nuclear Localization

**Figure 5:**
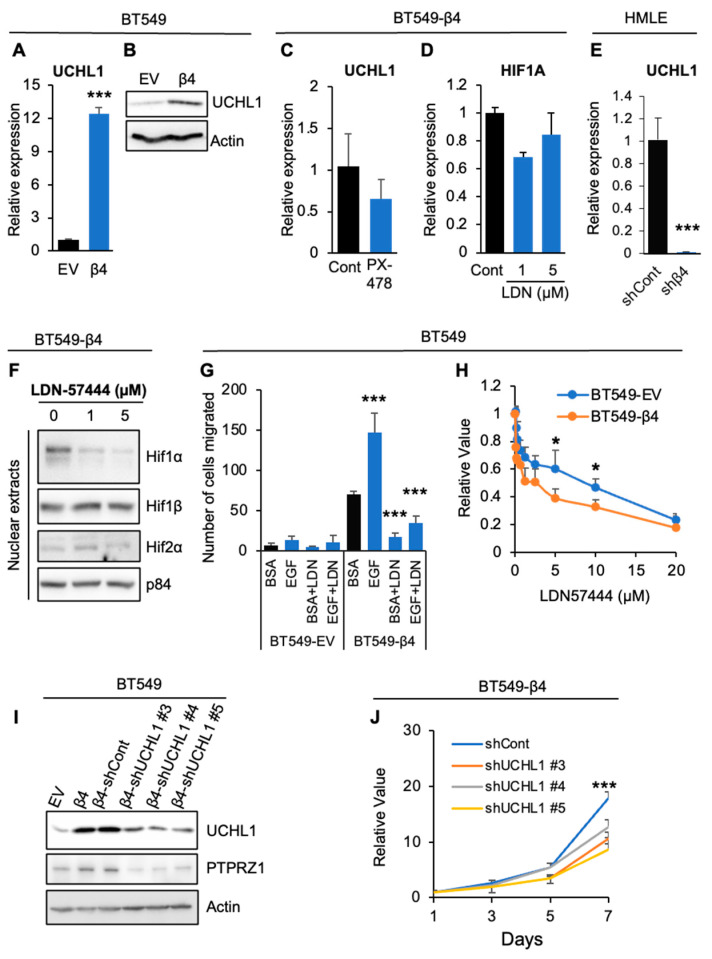
UCHL1 is critical for integrin α6β4-mediated Hif-1α nuclear accumulation and proliferation. (**A**,**B**) Expression of UCHL1 in BT549 cells (EV, β4) was assessed by qPCR (**A**) or immunoblotting (**B**). (**C**–**E**) BT549 β4 cells were treated with 10 µM PX-478 for 48 h (**C**) or indicated doses of LDN57444 (LDN) for 48 h. Cells were then harvested to assess the expression of UCHL1 (**C**) or HIF1A (**D**) by qPCR. (**E**) HMLE cells with integrin β4 shRNA knockdown (shβ4) or control shRNA (shCont) were used to assess the expression of UCHL1 by qPCR. (**F**) BT549 β4 cells as treated in (**D**) indicated proteins in nuclear extracts were evaluated by immunoblotting (**E**). (**G**) BT549 cells (EV, β4) were treated with 5 µM LDN57444 (LDN) for 1hr, and then assessed for cell migration toward BSA or 5 ng/mL EGF with the presence or absence of LDN for 3 h. (**H**) BT549-EV and -β4 cells were treated with different doses of LDN as indicated for 7 days, then MTT assays were performed. (**I**,**J**) BT549 (EV and β4) and BT549-β4 cells with UCHL1 stably knocked down using shRNA (**I**) were assessed for PTPRZ1 and UCHL1 by immunoblotting (**I**) or evaluated for cell proliferation using MTT (**J**). * *p* < 0.05, *** *p* < 0.0001.

We found that integrin β4 promotes Hif-1α nuclear accumulation without change at the mRNA level, suggesting that Hif-1α is stabilized by integrin α6β4 signaling. Notably, we found that UCHL1, which has been shown to regulate Hif-1α stability [19], was upregulated by integrin α6β4 at both mRNA and protein levels (Figure 5A,B). While UCHL1 is a hypoxia related gene, we found that UCHL1 is not a Hif-1α responsive gene, as inhibition of Hif-1α did not significantly change UCHL1 expression at the mRNA level (Figure 5C). Inhibition of UCHL1 also did not influence Hif-1α at the mRNA level (Figure 5D). Using HMLE cells with stable knockdown of integrin β4, we found that, compared to the shCont cells, ITGB4 knockdown cells dramatically decreased levels of UCHL1 (Figure 5E). To address whether integrin β4 upregulation of UCHL1 expression contributes to increased Hif-1α nuclear accumulation, we treated BT549-β4 cells with different doses of UCHL1 inhibitor LDN57444 for 48 h prior to immunoblot analysis of nuclear extracts. As shown in Figure 5F, inhibition of UCHL1 significantly reduced Hif-1α, but not the levels of Hif-1β and Hif-2α in the nucleus. To determine the role of UCHL1 in the migration and proliferation downstream of integrin α6β4, we treated BT549 EV and β4 cells with LDN-57444, and then assessed migration toward EGF in a Transwell migration assay and cell proliferation was determined by MTT assay. As shown in Figure 5G, while integrin β4 expression significantly promoted basal and EGF-stimulated migration compared to BT549 EV cells. inhibition of UCHL1 by LDN-57444 significantly decreased basal migration, as well as migration of BT549 β4 cells toward EGF. In contrast, treatment with LDN-57444 did not have significant impact on the migration for BT549-EV cells. When BT549 EV and β4 cells were treated with various doses of LDN57444, we found that inhibition of UCHL1 had greater impact on the proliferation of BT549 β4 cells when compared to BT549-EV cells (Figure 5H). Stable knockdown of UCHL1 in BT549 β4 cells by several shRNAs demonstrated a significant reduction in PTPRZ1 (Figure 5I), as well as decreased proliferation of BT549 β4 cells (Figure 5J). These data suggest that UCHL1 upregulation by integrin α6β4 is important for cell migration and proliferation and responsible for Hif-1α stabilization and upregulation of PTPRZ1.

### 3.4. Disruption of the UCHL1-Hif-1α-PTPRZ1 Axis Inhibits Integrin α6β4-Driven Migration and 3D Invasive Growth

**Figure 6:**
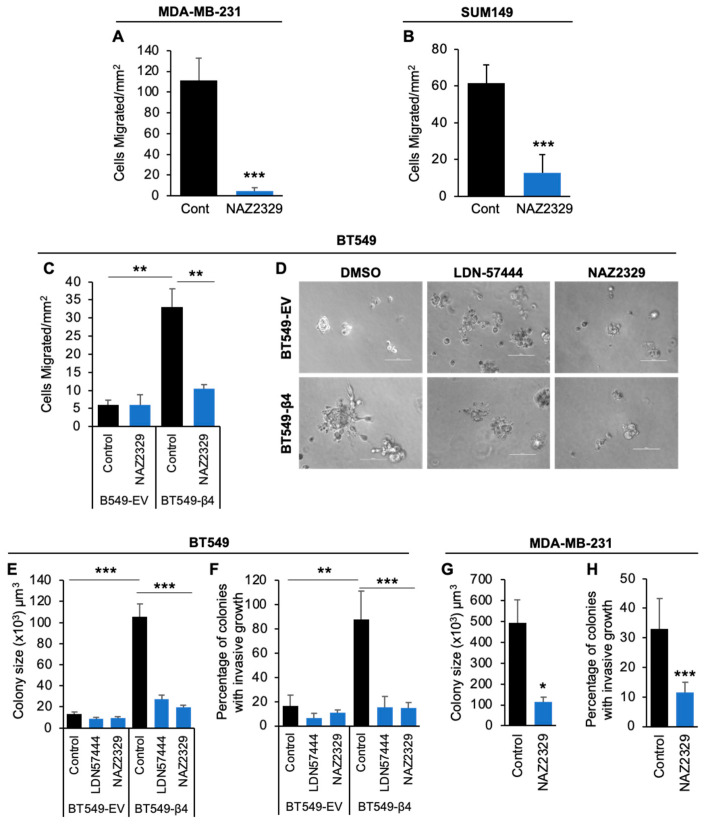
Inhibition of UCHL1 and PTPRZ1 abrogated integrin α6β4-driven invasive capacity. (**A**) MDA-MB-231), (**B**) SUM149, or (**C**) BT549 EV and BT549 β4 cells were treated with 10 µM NAZ2329 (PTPRZ1 inhibitor) for 24 h, then cell migration assays toward 5 ng/mL EGF were performed. (**D**–**F**) BT549 (EV and β4) cells were grown in 3D Matrigel in the presence of DMSO (Control), 5 µM LDN57444 (UCHL1 inhibitor), or 10 µM NAZ2329 for 5 days and then imaged (**D**), colony volume measured and calculated (**E**), and percentage of colonies with invasive growth determined (**F**). (**G**,**H**) MDA-MB-231 cells were grown in 3D Matrigel in the presence of DMSO (Control) or 10 µM NAZ2329 for 6 days and then colony size was calculated (**G**) and percentage of colonies with invasive growth was determined (**H**). Scale bars, 100 µm. * *p* < 0.05, ** *p* < 0.005, *** *p* < 0.0001.

We demonstrated the regulation of PTPRZ1 by Hif-1α and UCHL1 downstream of integrin α6β4, as well as the importance of UCHL1 in regulation of Hif-1α and cell migration. Next, we investigated how this signaling axis impacted the migratory and invasive capacity of cells downstream of integrin α6β4. We first treated MDA-MB- 231 (Figure 6A), SUM149 (Figure 6B), BT549 EV, and BT549-β4 cells (Figure 6C) with 10 µM of PTPRZ1 inhibitor NAZ2329 for 24 hrs and then performed cell migration assays toward EGF. We found that NAZ2329 treatment significantly abrogated the migratory capability of MDA-MB-231 (Figure 6A) and SUM149 (Figure 6B) cells toward EGF. While integrin β4 expression in BT549 cells increased cell migration as we have previously reported [20], NAZ2329 treatment dramatically decreased β4-promoted migration toward EGF but had no significant effect on BT549-EV cells (Figure 6C). Next, we tested the impact of inhibition of UCHL1 and PTPRZ1 in more physiologically relevant 3D culture conditions. Here, we cultured BT549 EV and BT549 β4 cells in 3D Matrigel with 2% FBS. We treated cells with 5 µM LDN57444 and 10 µM NAZ2339 in 3D for 7 days (Figure 6D), and then the size of the colonies was measured, volume calculated, and the invasive capacity assessed. As quantified in Figure 6E, integrin β4 increased BT549 cells grown in 3D, while inhibition of either UCHL1 or PTPRZ1 dramatically decreased the colony size of BT549 β4 cells. A minimum effect was noted on BT549 EV cells. Although more than 80% of colonies for BT549 β4 cells in 3D culture exhibited invasive growth, treatment with LDN57444 and NAZ2329 drastically decreased the percentage of colonies with invasive growth to approximately 20%, which is similar to BT549-EV cells (Figure 6F). Similar results were observed in MDA-MB-231 cells in 3D with NAZ2329 treatment (Figure 6G,H). Together, our data demonstrates the importance and critical roles of UCHL1 and PTPRZ1 in promoting the integrin α6β4-driven invasive phenotype of breast cancer cells.

### 3.5. High Expression Levels of Key Genes in the Integrin α6β4-PTPRZ1 Axis Correlate with Worse Outcomes for Breast Cancer Patients

**Figure 7:**
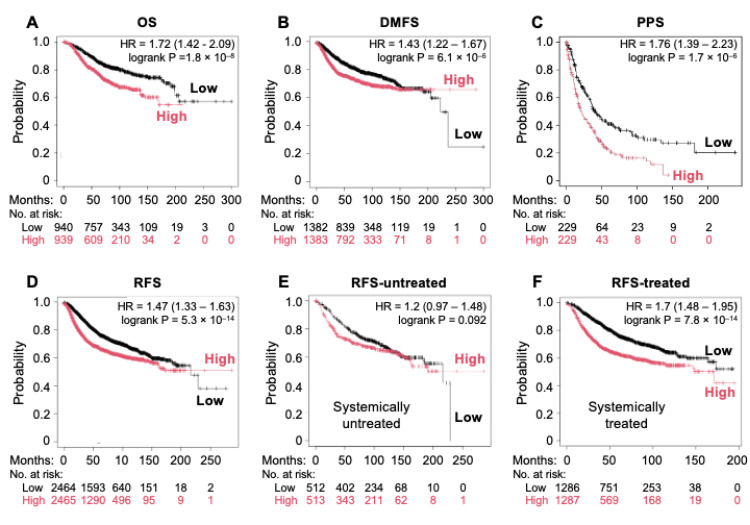
High expression levels of key genes in the integrin α6β4-PTPRZ1 axis correlates with worse outcomes for breast cancer patients. Using the Kaplan–Meier Plotter breast gene chip dataset, breast cancer patients were stratified into high and low expression groups based on mean expression of PTPRZ1, ITGB4, UCHL1, and HIF1A to assess OS (**A**), DMFS (**B**), PPS (**C**), and RFS (**D**). Patients with high versus low expression of these genes were further assessed against those receiving no systemic therapy (**E**) versus systemic therapy (**F**) to assess the impact on RFS. Log-rank *p*-values and hazard ratios were calculated in Kaplan–Meier Plotter.

Previous studies in breast cancer highlighted that PTPRZ1 was a risk factor for poor prognosis in TNBC and found that PTN-PTPRZ1 was driven by chemotherapy, indicating its role in chemoresistance [14] that gives rise to disease recurrence. We uncovered that the integrin α6β4-UCHL1-Hif-1α-PTPRZ1 signaling axis promoted an invasive phenotype, as discussed above, which is a property known to contribute to tumor progression. To determine the potential clinical relevance of our findings, we used the KM Plotter database breast gene chip dataset, which includes 7830 unique samples from 55 independent datasets from the GEO repository integrated into one dataset [25]. First, we assessed the prognostic value of the expression of individual genes in the integrin α6β4-UCHL1-Hif1α-PTPRZ1 pathway on survival outcomes in breast cancer. Based on the mean expression levels of ITGB4, UCHL1, HIF1A, and PTPRZ1, patients were divided into high and low expression groups using automatically selected optimal cutoff values. Survival analysis plots, hazard ratios, 95% confidence intervals, and log-rank *p*-values were calculated using KM Plotter. We plotted overall survival (OS, n = 1880), distant metastasis-free survival (DMFS, n = 2767), relapse-free survival (RFS, n = 4934), and post-progression survival (PPS, n = 458) for breast cancer in the high versus low expression groups. We found that, among these genes (Figure S2), high levels of HIF1A expression associate with statistically significant (log-rank *p*-value < 0.05) reduction in OS, DMFS, PPS, and RFS with HR of 1.58, 1.21, 1.84, and 1.39, respectively. UCHL1 was also associated with decreased DMFS (HR = 1.38). Interestingly, high expression levels of integrin β4 (ITGB4) or PTPRZ1 led to a modest increase in PFS with HR of 0.82 and 0.89, respectively, but not the other parameters assessed (Figure S2). When mean expression of PTPRZ1, ITGB4, UCHL1, HIF1A were evaluated together to assess the prognostic value of the integrin α6β4-UCHL1-HIF1A-PTPRZ1 axis in breast cancer, we found that, compared to the low expression group, high expression of these four genes associated with worse OS (HR = 1.72, Figure 7A), DMFS (HR = 1.43, Figure 7B), PPS (HR = 1.76, Figure 7C) and RFS (HR = 1.47, Figure 7D) for breast cancer patients with higher HR than HIF1A alone in all measures except PPS. We further stratified patients by those receiving systemic therapy (chemotherapy, endocrine therapy) versus no systemic therapy to assess the impact on RFS. Interestingly, we found that patients with high expression of these four genes exhibit similar RFS compared to low expressors in the group without systemic therapy (Figure 7E), but worse RFS with HR of 1.7 in the group with systematic treatments (Figure 7F). These data implicate an important role of Hif-1α-mediated PTPRZ1 expression though UCHL1 in integrin β4-driven breast cancer progression and patient outcomes.

## 4. Discussion

Our study highlights a novel function of integrin α6β4 signaling in the regulation of Hif-1α nuclear accumulation, which is required for the upregulation of PTPRZ1 expression. We also demonstrate that UCHL1 is involved in Hif-1α stability downstream of integrin α6β4, thus representing a novel activating mechanism for promoting Hif-1α signaling. Furthermore, gene ontology (GO) pathway enrichment analysis of RNA-seq data from cells with or without the β4 subunit uncovered enrichment of genes by integrin α6β4 that associate with hypoxia (Figure 3A), despite culturing in normoxia. Activation of Hif-1α under non-hypoxia conditions can be mediated by factors such as growth factors and oxidative stress, which are often mediated by metabolic stress that leads to the phenomenon of pseudohypoxia [26]. Hypoxic signatures that exist under normoxia or mild hypoxia can drive aggressive cancer properties, thus spawning the recent concept that pseudohypoxia can drive this hypoxia signature and TNBC tumor progression [26]. Under pseudohypoxia conditions, tumor cells use Hif-1α to promote invasion, metastasis, therapeutic resistance, and angiogenesis as adaptations to these stress conditions. Notably, both Hif-1α and Hif-2α are involved in pseudohypoxia. Although Hif-1α and Hif-2α are structurally related and share about 48% homology, they regulate distinct gene expression [26]. Our study demonstrated that integrin α6β4 confers a pseudohypoxia signature through Hif-1α. Interestingly, Hif-2α is decreased in BT549-β4 cells in the nucleus and transcriptional levels, suggesting that Hif-1α is the major contributor for integrin α6β4 to confer a pseudohypoxia signature in BT549-β4 cells. In contrast, we observed that, in MDA-MB-231 cells, integrin β4 KO clones displayed an opposite impact on Hif-2α nuclear accumulation. Given that TNBC is very heterogeneous and the complexity of this disease, the role of Hif-2α in integrin β4-driven pseudohypoxia signature could not be definitively ruled out. More models will be needed to further investigate if the regulation of PTPRZ1 by integrin α6β4-Hif-1α is universal in TNBC.

We find that integrin α6β4 signaling does not alter the Hif-1α mRNA level but consistently leads to Hif-1α nuclear accumulation. Hif-1α activity is impacted by the balance between degradation and stabilization. Under pseudohypoxia conditions, Hif-1α becomes stable through multiple mechanisms, resulting in Hif-1α nuclear translocation, in which Hif-1α heterodimerizes with Hif-1β, recruiting coactivators such as CBP/p300, to activate gene expression. Despite the ability of integrin α6β4 to regulate Hif-1α targets, integrin α6β1 has gained more attention as a regulator of Hif-1α, especially under hypoxia conditions [27]. A multitude of genes are regulated by Hif-1α in solid tumors, including VEGF. Previous studies have shown that integrin α6β4 dramatically upregulates VEGF expression in breast cancer through inactivation of translation factor inhibitor 4E-binding protein (4E-BP1) [28]. Although hypoxia can enhance eIF-4E expression, whether Hif-1α is also involved in the regulation of VEGF was not pursued. A recent study in gastric cancer showed that the extracellular matrix protein 1 directly interacts with integrin α6β4 and enhances Hif-1α promoter activity to regulate glucose metabolism through the FAK/Sox2 pathway [29], indicating that the regulation of Hif-1α by integrin α6β4-signaling is at multiple levels, and is not limited to TNBC. Here, we expand on these studies to find that integrin α6β4 can stabilize Hif-1α through UCHL1 in BT549 β4 cells under non-hypoxia conditions, and that this stabilization ultimately leads to the upregulation of PTPRZ1. Since PTPRZ1 is part of the pseudohypoxia signature regulated by integrin α6β4, these observations suggest that integrin α6β4, through its sensing of the tumor microenvironment, could regulate pseudohypoxia to promote tumor progression.

PTPRZ1 is regulated by both chronic oxidative stress and hypoxia. PTPRZ1 was also identified as a responsive gene of Hif-2α [23,24] and MIR1261/cir-PTP [10]. While integrin α6β4 in TNBC cells regulates PTPRZ1 through Hif-1α, we found that activating Hif-1α using an activator is not sufficient to regulate PTPRZ1 expression without integrin β4 (BT549 EV), suggesting that other factors regulated by integrin β4 have to cooperate with Hif-1α to regulate PTPRZ1 expression. Notably, previous studies showed that several transcription factors, such as NF-κB and ZEB1, which are downstream of integrin α6β4 also regulate PTPRZ1 in several systems [10]. Given that Hif-1α cooperates with other transcription factors and integrin α6β4 can epigenetically regulate gene expression, it will be interesting to further investigate whether PTPRZ1 is regulated by the cooperation of Hif-1α with other transcription factors, or in conjunction with epigenetic events such as DNA methylation and histone methylation downstream of integrin α6β4, in future studies.

While our study discovered a significant correlation of ITGB4 and PTPRZ1 in various cancer types, including TNBC, it is worth mentioning that ITGB4 also significantly correlated with PTPRZ1 in luminal breast cancer subtype. PTPRZ1 has several ligands, such as PTN and midkine. Interestingly, BT549 cells express these ligands and some of these ligands can bind integrins. For example, midkine can bind integrin α4β1 and α6β1. At this stage, it is not clear whether integrin promotes the expressions of these ligands. Since PTN and PTPRZ1 are both expressed in different subtypes of breast cancer and PTN is also found in normal breast tissues [30,31], it will be interesting to investigate how the correlation of ITGB4 and PTPRZ1 contributes to luminal cancer progression and therapeutic response. Likewise, we also found that integrin α6β4 regulation of PTPRZ1 exists in HMLE normal mammary epithelial cells, suggesting that the integrin α6β4-PTPRZ1 axis plays fundamental roles in mammary cells, which needs to be further investigated.

PTPRZ1 is best known for its role in axonal guidance and contributions to stem cell properties in glioblastoma [11], and blocks oligodendrocyte differentiation [32]. PTPRZ1 also has many substrates, including p190RhoGAP and β-catenin, which contribute to tumor progression [11]. Notably, P190RhoGAP can promote the switch of active GTP-bound Rho into the GDP-bound inactive form, an important signaling protein downstream of integrins, which is known to promote cell migration and invasion [33]. Determining which substrate is critical has the potential to be beneficial to the development of better treatment options for TNBC patients.

Our data demonstrated that disruption of the integrin α6β4-UCHL1-Hif-1α-PTPRZ1 pathway through UCHL1 and PTPRZ1 inhibitors significantly abrogated α6β4-mediated invasive growth and migration. These results suggest that both UCHL1 and PTPRZ1 represent viable targets for TNBC treatment. We also found that high expression levels of the key proteins in the pathway (integrin β4, UCHL1, Hif-1α, and PTPRZ1) correlated with poorer patient survival outcomes. Interestingly, patients with systematic treatment who have high expression of the four genes have the worst RFS compared to patients without systematic treatment, suggesting that this pathway also impacts outcomes of treatment in patients with breast cancer. Previous studies in breast cancer highlighted that PTPRZ1 is a risk factor for poor prognosis in TNBC and found that PTN-PTPRZ1 is driven by chemotherapy, indicating its role in chemoresistance. PTPRZ1 is increased in both clinical samples and cell lines, in response to doxorubicin through the CDKN1A-NF-κB pathway [14]. In this regard, our study bears several interesting clinical implications. First, this pathway could contribute to the stemness of cancer cells, which impacts tumor progression and therapeutic response. Secondly, elevated expression levels of integrin β4 associated with the aggressive features of TNBC by promoting EMT, invasion, and metastasis. Given the normal function of integrin β4 in maintaining the integrity of epithelium, it is not practical to directly target integrin β4 itself. Our study suggests that targeting the integrin α6β4-PTPRZ1 axis offers a potential therapeutic strategy to block integrin β4 signaling-driven invasive capacity in TNBC, which could be further investigated using the TNBC in vivo models. Third, a previous study showed that integrin α6β4 associated with cancer stem cells and negatively correlated with TNBC relapse-free survival in which PTPRZ1 shares the same features. Elevated expression levels of four proteins in the integrin α6β4-UCHL1-HIF1A-PTPRZ1 axis, in particular, negatively associated with RFS for breast cancer patients with treatment, suggesting that the expression signature of these four genes has prognostic value for treatment of breast cancer patients.

## 5. Conclusions

**Figure 8:**
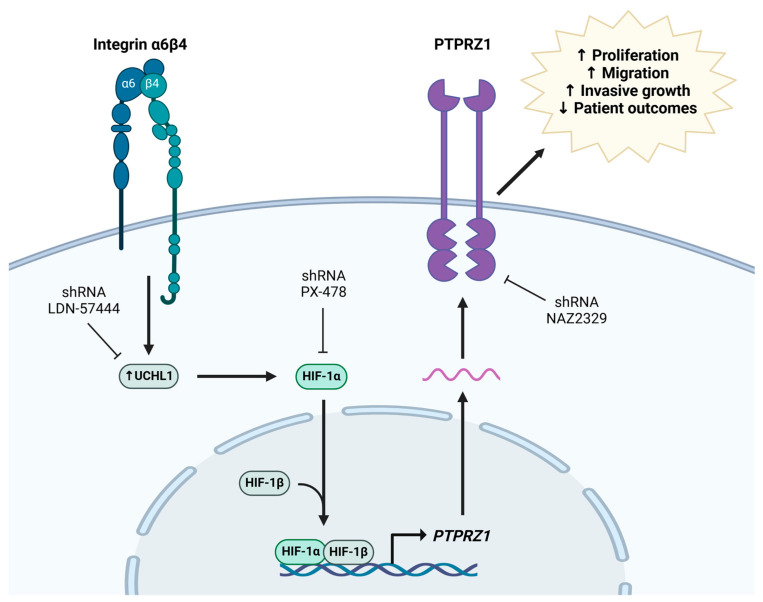
Proposed signaling downstream of integrin α6β4 that promotes invasive capacity through the UCHL1-Hif-1α-PTPRZ1 pathway.

Our study reveals that integrin α6β4 plays a critical role in promoting the aggressiveness of TNBC by upregulating the expression of the protein tyrosine phosphatase PTPRZ1 through UCHL1-mediated Hif-1α signaling (Figure 8). Our findings demonstrate that dysregulating this pathway contributes to the invasive phenotype of TNBC, with high expression levels of integrin β4, UCHL1, Hif-1α, and PTPRZ1 correlating with poorer patient survival outcomes. Thus, our study suggests that targeting integrin β4 signaling through the inhibition of PTPRZ1 and UCHL1 could represent a novel therapeutic approach for treating TNBC.

## Figures and Tables

**Table 1:** Correlation of ITGB4 and PTPRZ1 in breast cancer.

Breast Cancer Subtypes	Spearman Correlation	*p* Value
Basal	0.23	2.17 × 10^−3^
Luminal A	0.35	1.13 × 10^−15^
Luminal B	0.05	0.51
HER2	0.1	0.397

Using TCGA PanCancer Atlas genomic data, the Spearman correlations between ITGB4 and PTPRZ1 expression within breast cancer subtypes were calculated in cBioPortal.

## Data Availability

The original contributions presented in the study are included in the article/Supplementary Materials, further inquiries can be directed to the corresponding authors.

## Supplementary Materials

The following supporting documents can be downloaded at: https://www.mdpi.com/article/10.3390/cancers16213683/s1, Figure S1: TCGA database analysis for PTPRZ1 expression in different cancer types; Table S1: Correlation of ITGB4 and PTPRZ1 in selected cancers; Figure S2: Individual survival plots of ITGB4, UCHL1, HIF1A, and PTPRZ1 in breast cancer.
