# Supplementary material for: Integrin α6β4 Upregulates PTPRZ1 Through UCHL1-Mediated Hif-1α Nuclear Accumulation to Promote Triple-Negative Breast Cancer Cell Invasive Properties
Source: Cancers (Basel). 2024 Oct 31;16(21):3683. doi: 10.3390/cancers16213683 (PMC11545476; doi:10.3390/cancers16213683)
Supplement: Supplementary file 1 [file cancers-16-03683-s001.zip › cancers-3241092-supplementary.pdf]

**SUPPLEMENTAL MATERIALS**

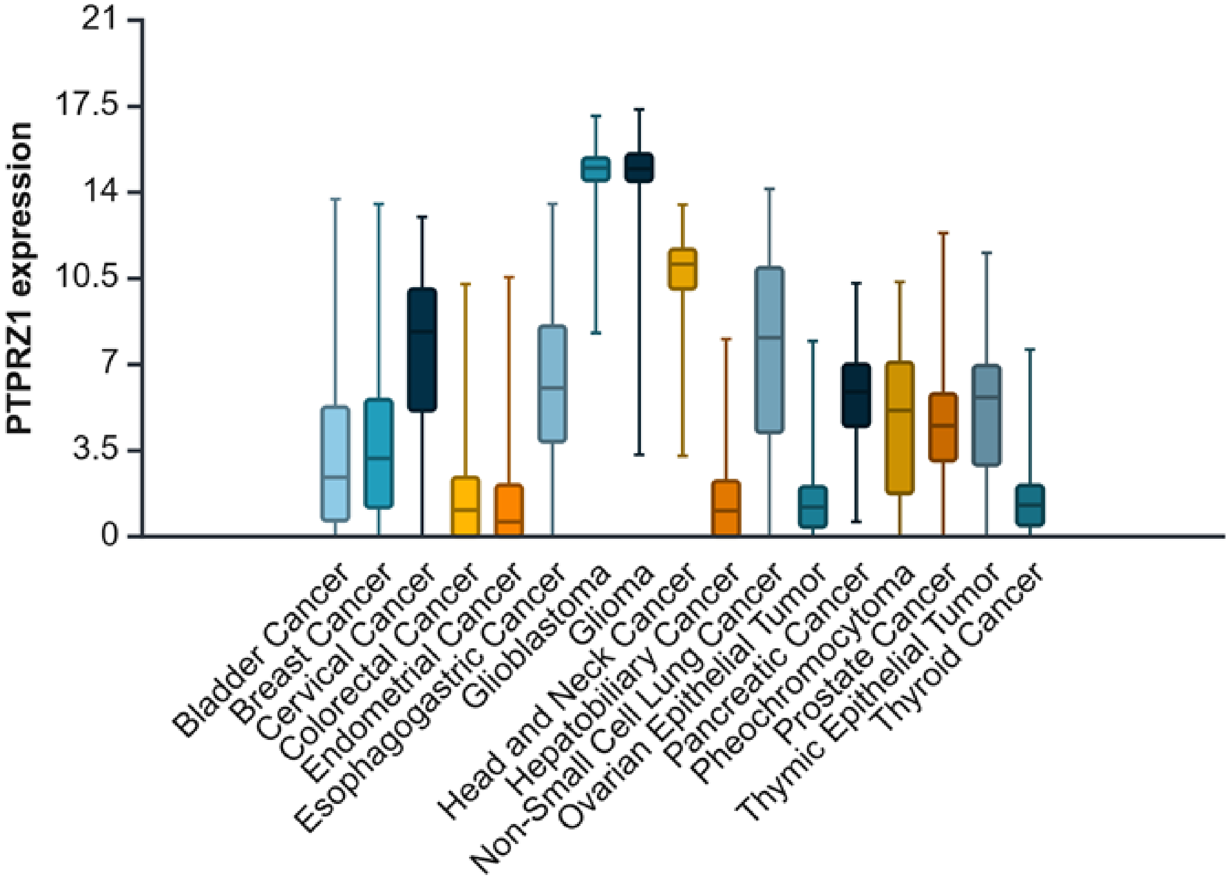


**Figure S1.** TCGA database analysis for PTPRZ1 expression in different cancer types. Using TCGA PanCancer Atlas data from cBioPortal, PTPRZ1 mRNA expression data from representative cancers was plotted by cancer type in BioRender (log2(value+1), RSEM, Batch normalized from Illumina HiSeq_RNASeqV2).

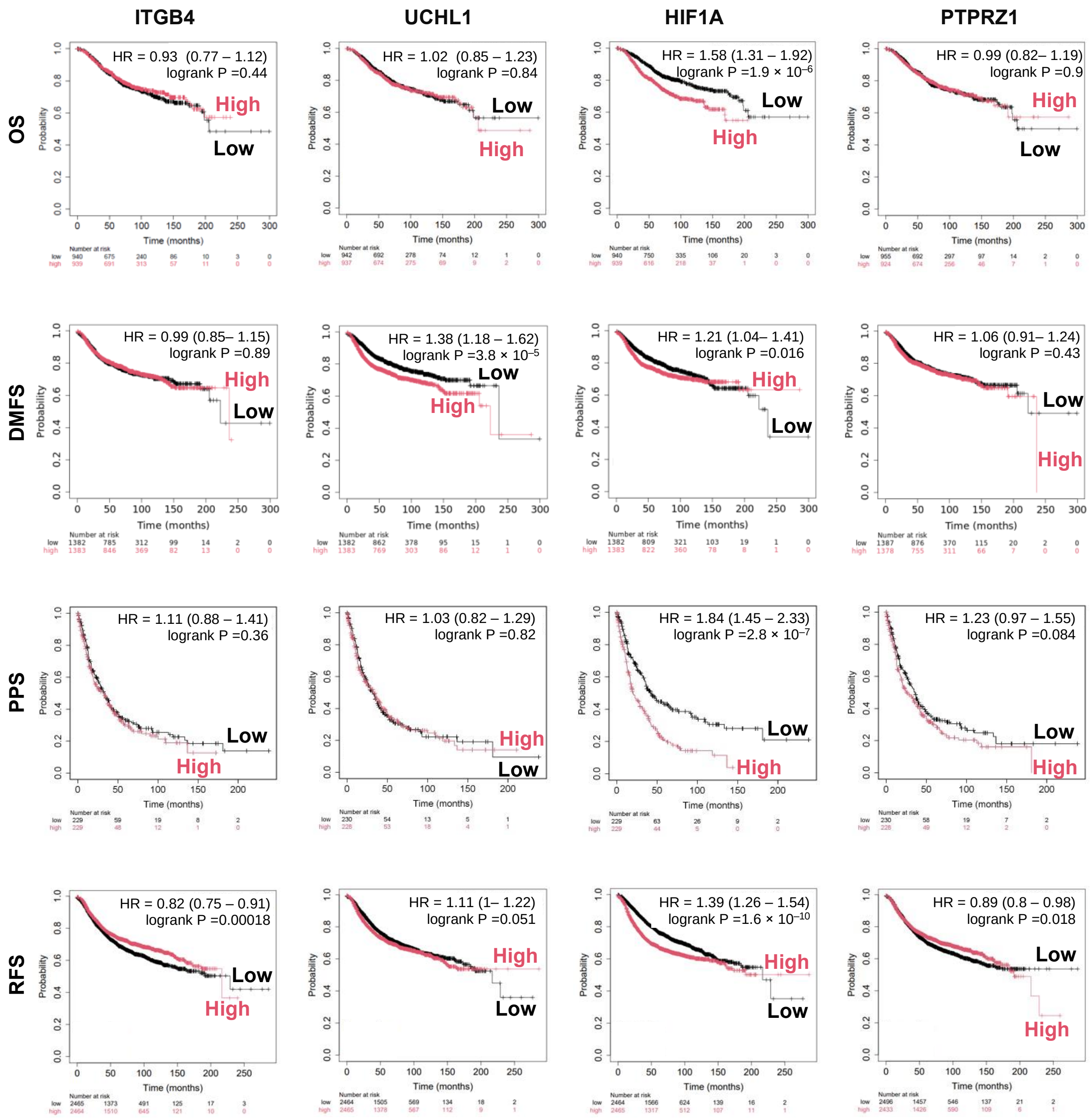


**Figure S2.** Individual survival plots of ITGB4, UCHL1, HIF1A, and PTPRZ1 in breast cancer. Using the Kaplan–Meier Plotter breast gene chip dataset, breast cancer patients were stratified into high and low expression groups based on mean expression of ITGB4, UCHL1, HIF1A, and PTPRZ1 individually to assess overall survival (OS), distant metastasis-free survival (DMFS), post-progresson survival (PPS), and relapse-free survival (RFS). Logrank p values and hazard ratios were calculated in Kaplan–Meier Plotter.

**Table S1. Correlation of ITGB4 and PTPRZ1 in selected cancers.**

| Cancer type | Speraman Correlation | p value |
|---|---|---|
| Pheochromocytoma & paraganglioma | 0.65 | $1.25\times10^{-20}$ |
| Prostate cancer | 0.63 | $7.86\times10^{-55}$ |
| Lung cancer | 0.46 | $6.10\times10^{-53}$ |
| Thymoma | 0.44 | $7.13\times10^{-7}$ |
| Breast Cancer | 0.36 | $5.76\times10^{-31}$ |
| Liver hepatocellular carcinoma | 0.34 | $5.64\times10^{-11}$ |
| Esophageal adenovarcinoma | 0.32 | $1.17\times10^{-5}$ |
| Lung squamous cell carcinoma | 0.22 | $1.77\times10^{-6}$ |
| Glioblastoma multiforme | -0.26 | $1.45\times10^{-3}$ |
| Brain lower grade glioma | -0.35 | $2.59\times10^{-16}$ |

Using TCGA PanCancer Atlas genomic data, the Spearman correlations between ITGB4 and PTPRZ1 expression across multiple cancer types were calculated in cBioPortal.
